# Fruit and Vegetable Consumption and Inflammatory Bowel Disease: A Case-Control Study

**DOI:** 10.3390/life14121524

**Published:** 2024-11-21

**Authors:** Anas M. Almofarreh, Haytham A. Sheerah, Ahmed Arafa, Fairuz A. Algori, Ghonem R. Almutairi, Kafi A. Alenzi, Madiha M. Al-Alsehemi, Banan H. Mekwar, Osama Alzeer, Haneen N. Molla

**Affiliations:** 1 Assistant Deputyship for Health Investment Development, Ministry of Health, Riyadh 11451, Saudi Arabia; aalmofarreh@moh.gov.sa; 2 Office of the Vice Minister of Health, Ministry of Health, Riyadh 11451, Saudi Arabia; 3 Department of Preventive Cardiology, National Cerebral and Cardiovascular Center, Suita 564-8565, Japan; ahmed011172@med.bsu.edu.eg; 4 Department of Public Health and Community Medicine, Faculty of Medicine, Beni-Suef University, Beni-Suef 62521, Egypt; 5 Assistant Deputyship for Preventive Health, Ministry of Health, Riyadh 11451, Saudi Arabia; 6 General Administration for Health Facilities Licensing, Ministry of Health, Riyadh 11451, Saudi Arabia; gralmotari@moh.gov.sa; 7 Assistant Deputyship for Medical Assistance Services, Ministry of Health, Riyadh 11451, Saudi Arabia; 8 Department of Legislation and Regulations, General Directorate of Nursing and Midwifery Affairs, Ministry of Health, Riyadh 11451, Saudi Arabia; 9 Deputyship for Therapeutic Services, Ministry of Health, Riyadh 11451, Saudi Arabia; bmekwar@moh.gov.sa; 10 Department of Clinical Nutrition, King Khalid University Hospital, King Saud University Medical City, Riyadh 12372, Saudi Arabia

**Keywords:** Crohn’s disease, diet, food, observational study, ulcerative colitis, vegetables

## Abstract

Background: Inflammatory bowel diseases (IBD), including ulcerative colitis (UC) and Crohn’s disease (CD), are chronic conditions with complex etiologies that may involve dietary factors. This study investigates the association between fruit and vegetable consumption and the risk of UC and CD, focusing on gender-specific differences. Methods: A hospital-based case–control study, comprising 158 UC patients (93 men and 65 women), 245 CD patients (167 men and 78 women), and 395 controls without IBD (256 men and 139 women), was conducted in Riyadh, Saudi Arabia. Fruit and vegetable consumption data were gathered through a self-administered questionnaire distributed before diagnosis. Logistic regression analysis was applied to estimate the odds ratios (ORs) and 95% confidence intervals (95% CIs) for UC and CD among the participants reporting the daily consumption of fruits and vegetables. Results: Among men, daily vegetable consumption was associated with higher odds of UC in the age-adjusted model [OR (95% CI): 1.78 (1.02, 3.10)], but this association became non-significant after further adjustment for body mass index, smoking, anemia, and elevated liver enzymes [OR (95% CI): 1.70 (0.91, 3.18)]. No significant associations were observed between vegetable consumption and CD. In contrast, the women who consumed vegetables every day had a non-significant inverse association with UC and a significant inverse association with CD in both the age-adjusted and multivariable-adjusted models [ORs (95% CIs): 0.44 (0.23, 0.87) and 0.41 (0.20, 0.84), respectively]. Fruit consumption was neither associated with UC nor CD in either sex. Conclusions: Daily vegetable consumption was significantly associated with decreased odds of CD among women, but not men, highlighting potential sex-specific dietary influences on IBD risk.

## 1. Introduction

Inflammatory bowel disease (IBD) refers to a group of chronic, relapsing inflammatory conditions of the gastrointestinal tract, primarily including ulcerative colitis (UC) and Crohn’s disease (CD). Both conditions are characterized by chronic inflammation, which may affect various parts of the digestive tract, leading to symptoms such as abdominal pain, diarrhea, and fatigue. Despite extensive research, the exact etiology of IBD remains unclear, but it is believed to result from a complex interaction between genetic, environmental, and immune factors [1].

The burden of IBD is substantial, affecting millions of individuals worldwide, with rising incidence rates, particularly in newly industrialized regions [2,3]. Between 1990 and 2019, the age-standardized prevalence rate of IBD increased in 13 out of the 21 Global Burden of Disease (GBD) regions, and by 2019, there were approximately 4.9 million IBD patients worldwide [2]. In countries with historically low IBD prevalence, including those in the Arab region, an increase in cases has been observed over recent decades, likely driven by urbanization and the Westernization of diets. A meta-analysis of 16 studies investigating Arab populations put UC and CD incidences at 2.33 and 1.46 per 100,000 person–years, respectively [4]. In Saudi Arabia, where the rapid shift toward a Western lifestyle has been pronounced, IBD represents an emerging public health challenge [5], but limited data exist on its risk factors within the local population.

Diet has long been suggested as a key modifiable factor in the development and progression of IBD. Emerging evidence has highlighted the potential role of fruits and vegetables in influencing IBD risk [6,7], given their rich content of fiber, antioxidants, vitamins, and anti-inflammatory properties [8]. However, a few studies have investigated the association between fruit and vegetable consumption and IBD risk and even reached inconsistent conclusions. In a population-based case–control study involving 256 UC patients, 186 CD patients, and 940 controls from eight Asian countries and Australia, fruit and vegetable consumption was not associated with UC and CD [9]. In a prospective cohort study using data from the US Nurses’ Health Study II (103 UC and 70 CD incident cases), women who reported a high-school diet, involving greater amounts of fruits, vegetables, and fish, had a lower risk of CD, but not UC [10]. In a case–control study, including 51 UC patients, 81 CD patients, and 104 controls from Australia, the daily consumption of fruits was negatively associated with UC but not CD, while the daily consumption of vegetables was neither associated with UC nor CD [11]. In another case–control study involving 144 UC patients, 123 CD patients, and 267 controls from Denmark, the daily consumption of fruits and vegetables was associated with lower odds of UC and CD [12].

In addition, there is a significant gap in the knowledge about the association between dietary factors and IBD among Arab populations. Most studies have been conducted in Western countries, and it is unclear whether these findings apply to populations in different sociocultural contexts, such as Saudi Arabia. In addition, although sex-based differences in IBD pathogenesis, risk factors, disease course, and response to therapy have been documented [13], previous studies did not highlight the sex-specified associations between dietary factors and IBD.

In this context, this study aims to investigate the association between fruit and vegetable consumption and the risk of UC and CD among men and women in Saudi Arabia.

## 2. Methods

### 2.1. Study Population

The sample size calculation for this study has been detailed elsewhere [14]. The study population was drawn from individuals, aged ≥18 years, diagnosed at a private polyclinic in Riyadh, Saudi Arabia between January 2009 and December 2017. This case–control study included 171 patients with UC, 251 with CD, and 400 individuals with other gastrointestinal conditions, who acted as the control group. Eligibility for the UC and CD groups required a recent diagnosis, while the control participants needed to be free from IBD, malignancy, polyposis, and diverticulosis. The participants lacking fruit and vegetable consumption data were excluded, resulting in a final sample of 158 UC patients, 245 CD patients, and 395 controls for the statistical analysis.

### 2.2. Diagnosis of Ulcerative Colitis and Crohn’s Disease

As outlined in previous studies [14,15,16], the participants presenting with symptoms indicative of IBD, including abdominal pain, diarrhea, bloating, loss of appetite, unexplained weight loss, or bloody stool, underwent laboratory testing. These tests included urine and stool analyses for inflammatory biomarkers, as well as blood tests to measure the complete blood count, C-reactive protein, the erythrocyte sedimentation rates, bilirubin, alanine aminotransferase, creatinine, and the alkaline phosphatase levels. The participants with both clinical symptoms and laboratory results consistent with IBD were further evaluated through gastrointestinal endoscopy. A histopathological examination of the biopsy samples was then performed to confirm the diagnosis.

### 2.3. Assessment of Fruit and Vegetable Consumption

Data on fruit and vegetable consumption were gathered through a self-administered questionnaire distributed before ascertaining the IBD diagnosis. To evaluate their typical weekly consumption, the participants were asked, “How often do you eat fruits?” and “How often do you eat vegetables?” The response options for both questions were “once per month or less”, “once per week”, “twice per week”, “every day”, “do not remember” and “NA”. For statistical analysis, the responses “once per month or less”, “once per week”, and “twice per week” were combined into a single category labeled “infrequent consumption”. The participants who selected “do not remember” or “NA” were excluded from the analysis. The illiterate participants received assistance from data collectors.

### 2.4. Statistical Analysis

Logistic regression was used to compute the odds ratios (ORs) and 95% confidence intervals (CIs) for the UC and CD of frequent versus infrequent fruit and vegetable consumption. The results were adjusted for age only in Model I and were further adjusted for body mass index (BMI), smoking, anemia, and elevated liver enzymes in Model II. Since sex interacted with the association between fruit and vegetable consumption and IBD (*p*-values for sex interactions were <0.10), all the analyses were stratified by sex. Age and BMI, on the other hand, showed no interaction (*p*-values for age and BMI interactions were >0.20). The Statistical Package for Social Science (SPSS) 2013 (IBM SPSS Statistics for Windows, Version 22.0, IBM Corporation, Armonk, NY, USA) was used for data analysis.

## 3. Results

Among the 516 men in the study, 93 were diagnosed with UC, 167 with CD, and 256 served as controls. The men with UC were significantly older than those in the CD and control groups; 47.3% of the UC patients were aged ≥40 years, compared to 25.2% of the CD patients and 34.0% of the controls. The proportion of individuals with a BMI ≥ 25 kg/m^2^ was lower among the UC and CD patients compared to the controls, with 39.0% in UC, 27.5% in CD, and 57.4% in the control group. Additionally, the control group had a higher proportion of current smokers (32.0%) than the UC (18.3%) and CD (26.9%) groups. While the proportion of daily fruit consumption did not significantly differ across the groups—11.8% in UC, 15.0% in CD, and 14.1% in the controls—the UC patients reported more daily vegetable consumption (73.1%) compared to the CD patients (55.1%) and the controls (59.8%). The prevalence of anemia also varied significantly, with 11.7% in the controls, 47.3% in the UC patients, and 18.0% in the CD patients (Table 1).

Among the 282 women, 65 were diagnosed with UC, 78 with CD, and 139 served as controls. The women with CD were significantly younger than those with UC and the controls; 42.3% of the CD patients were <30 years old, compared to 21.5% of the UC patients and 18.0% of the controls. The proportion of women with a BMI ≥ 25 kg/m^2^ was lower in the CD group compared to the UC and control groups: 35.9% in CD, 60.3% in UC, and 63.3% in the controls. While the proportion of daily fruit consumption did not significantly vary across the groups, 13.8% in UC, 11.5% in CD, and 12.9% in the controls, the UC and CD patients reported less daily vegetable consumption (66.2% and 50.0%, respectively) compared to the controls (79.1%). Anemia was less common in the control group (22.3%) compared to the UC (38.5%) and CD (30.8%) groups (Table 1).

In the age-adjusted and multivariable-adjusted regression models, daily fruit consumption was not associated with UC or CD in either sex (Table 2). Among men, daily vegetable consumption was associated with increased odds of UC in the age-adjusted model [OR (95% CI): 1.78 (1.02, 3.10)]; however, this association lost statistical significance after further adjustments [OR (95% CI): 1.70 (0.91, 3.18)]. No significant associations were found between vegetable consumption and CD (Table 3). In women, daily vegetable consumption was inversely associated with UC in the age-adjusted model, despite not reaching statistical significance [OR (95% CI): 0.50 (0.23, 1.07)], and the association remained similar in the multivariable-adjusted model [OR (95% CI): 0.48 (0.22, 1.06)]. Daily vegetable consumption was significantly associated with lower odds of CD in both the age-adjusted and multivariable-adjusted models [ORs (95% CIs): 0.44 (0.23, 0.87) and 0.41 (0.20, 0.84), respectively] (Table 3).

## 4. Discussion

In this case–control study, we investigated the association between fruit and vegetable consumption and the risk of developing UC and CD, in a population from Riyadh, Saudi Arabia. Our findings suggest that the women, but not men, who reported higher vegetable consumption showed a reduced risk of IBD, particularly UC. No significant association was observed between fruit consumption and IBD risk in either sex. These results highlight the potential protective role of vegetables in reducing IBD risk but indicate that fruit consumption might not have the same effect in this population. They also highlight the sex-based differences in the dietary factors related to IBD.

The observed protective effect of vegetables may be explained by their rich fiber content, antioxidants, vitamins, and anti-inflammatory compounds. Vegetables, particularly leafy greens, are high in insoluble fiber, which promotes gut health by improving bowel regularity, enhancing microbial diversity, and producing short-chain fatty acids (SCFAs), such as butyrate. SCFAs have anti-inflammatory effects and play a crucial role in maintaining the intestinal barrier function, which is often compromised in IBD [8,17,18]. Additionally, the antioxidants found in vegetables, including vitamins A, C, and E, help neutralize oxidative stress, a key contributor to the chronic inflammation observed in IBD [19,20]. In contrast, the lack of a significant association between fruit consumption and IBD risk could be attributed to fruits’ fermentable sugars, such as fructose, which can lead to gastrointestinal symptoms, particularly in individuals with gut inflammation [21]. These sugars may disrupt gut microbiota balance, potentially counteracting any protective effects from fiber or antioxidants.

The sex-based differences in the nutritional factors related to IBD are not well-documented [13]; however, the differences in our study could be attributed to the dietary behaviors across the sexes. Women often consume more vegetables than men [22,23]. This higher intake could lead to a more obvious protective effect in women, while men might not consume enough vegetables to experience the same benefit.

Of note, a meta-analysis of observational studies showed that fruit consumption (measured as g/day in three studies) was negatively associated with UC and CD [ORs (95% CIs): 0.69 (0.55, 0.86) and 0.47 (0.38, 0.58), respectively]. Similarly, fruit consumption (measured as g/day) was negatively associated with UC and CD [ORs (95% CIs): 0.56 (0.48, 0.66) and 0.52 (0.46, 0.59), respectively] [24]. When the meta-analysis investigated the associations with additional servings of fruits and vegetables, fruit and vegetable consumption was negatively associated with CD. In contrast, the association with UC did not reach statistical significance. It is also worth mentioning that all the sub-analyses of this meta-analysis showed moderate to high heterogeneity across the studies [24].

Beyond their role in IBD development, the impact of fruit and vegetable consumption on IBD activation has shown mixed results. One study found that consuming both leafy and non-leafy vegetables, as well as fruits, exacerbated the IBD symptoms [25]. In contrast, a more recent study reported that fruit and vegetable intake reduced the prevalence of active IBD by 44% and helped prevent remission [26].

From the perspective of preventive medicine, our findings have important clinical and public health implications since they reinforce the role of a vegetable-rich diet in maintaining gut health and preventing inflammatory conditions like IBD. Clinicians and public health professionals should encourage higher vegetable consumption as part of the dietary guidelines aimed at reducing IBD risk, especially in regions like Saudi Arabia, where the incidence of IBD is rising. Since vegetables appear to offer protective effects against inflammation in the gut, integrating them into daily diets may serve as a cost-effective, preventive measure. On a clinical level, dietary interventions focusing on increasing vegetable intake could be considered as part of a holistic management approach for patients at high risk of IBD or those in the early stages of the disease.

This study presents several strengths, such as investigating an understudied population and utilizing standardized diagnostic methods for UC and CD. However, several limitations should be acknowledged. First, the data were collected from a single polyclinic in Riyadh. Since this is a private institution, the patients likely represent a higher socioeconomic group. This may limit the generalizability of our results. Second, recruitment occurred over an extended period, during which dietary habits and trends related to fruit and vegetable consumption may have shifted. Third, the food frequency questions were neither validated nor pre-tested. Fourth, a limitation of this questionnaire is that it assessed fruit and vegetable consumption in aggregate rather than individually, without specifying the types of fruits and vegetables consumed. In addition, the study did not account for the specific components of the fruits and vegetables (e.g., fiber, antioxidants, and vitamins), which could have varying effects on IBD risk. This approach may overlook variations in nutrient content and the health benefits associated with different fruits and vegetables. Fifth, due to the limited number of cases in each fruit and vegetable consumption frequency group, we combined the non-daily consumers into one group. This precluded the analysis of a potential dose–response relationship. Finally, we did not adjust our results for important confounders, such as total caloric intake. Seventh, our control group comprised patients with other gastrointestinal conditions. This may have led to an underestimation of the association between fruit and vegetable consumption and IBD due to overlapping dietary patterns.

## 5. Conclusions

This study found a negative association between daily vegetable consumption and the risk of CD among women but not men, suggesting that a higher intake of vegetables may have a protective effect against CD in Arab women. In addition, daily vegetable consumption showed a tendency of a protective effect against UC risk in women but not men. These findings highlight the importance of considering gender-specific dietary patterns in the prevention of IBD. On the other hand, no similar associations were observed with fruit consumption, indicating that the protective dietary factors against IBD may be more specific to vegetable intake. Further research, especially with larger cohorts, diverse populations, and prospective cohort designs, is needed to confirm these associations and to explore the underlying mechanisms.

## Figures and Tables

**Table 1:** Comparison between cases and controls in the case–control study.

Characteristics		Ulcerative Colitis	Crohn’s Disease	Control
Men (*n* = 516)				
Number of participants		93	167	256
Age, years, %	<30	19.4	29.3	18.4
	30–39	33.3	45.5	47.6
	≥40	47.3	25.2	34.0
Body mass index, kg/m^2^, %	<18.5	7.5	17.4	9.4
	18.5–24.9	53.8	55.1	33.2
	25.0–29.9	21.5	16.8	27.7
	≥30	17.2	10.7	29.7
Fruit consumption, %	Daily	11.8	15.0	14.1
	Infrequent	88.2	85.0	85.9
Vegetable consumption, %	Daily	73.1	55.1	59.8
	Infrequent	26.9	44.9	40.2
Current smoking, %		18.3	26.9	32.0
Anemia, %		47.3	18.0	11.7
Elevated liver enzymes, %		4.3	6.6	9.8
Women (*n* = 282)				
Number of participants		65	78	139
Age, years, %	<30	21.5	42.3	18.0
	30–39	36.9	41.0	33.1
	≥40	41.6	16.7	48.9
Body mass index, kg/m^2^, %	<18.5	9.8	20.5	9.4
	18.5–24.9	29.9	43.6	27.3
	25.0–29.9	27.5	26.9	28.8
	≥30	32.8	9.0	34.5
Fruit consumption, %	Daily	13.8	11.5	12.9
	Infrequent	86.2	88.5	87.1
Vegetable consumption, %	Daily	66.2	50.0	79.1
	Infrequent	33.8	50.0	20.9
Current smoking, %		1.5	1.3	0.0
Anemia, %		38.5	30.8	22.3
Elevated liver enzymes, %		7.7	7.7	4.3

**Table 2:** Association between daily fruit consumption and inflammatory bowel disease.

	Model I	Model II
Men		
Control	1 (Reference)	1 (Reference)
Ulcerative colitis	0.72 (0.34, 1.50)	0.99 (0.45, 2.22)
Crohn’s disease	1.28 (0.72, 2.27)	1.30 (0.71, 2.39)
Women		
Control	1 (Reference)	1 (Reference)
Ulcerative colitis	1.15 (0.48, 2.76)	1.23 (0.50, 3.02)
Crohn’s disease	1.31 (0.52, 3.32)	1.27 (0.48, 3.33)

**Table 3:** Association between daily vegetable consumption and inflammatory bowel disease.

	Model I	Model II
Men		
Control	1 (Reference)	1 (Reference)
Ulcerative colitis	1.78 (1.02, 3.10)	1.70 (0.91, 3.18)
Crohn’s disease	1.01 (0.66, 1.54)	1.02 (0.66, 1.60)
Women		
Control	1 (Reference)	1 (Reference)
Ulcerative colitis	0.50 (0.23, 1.07)	0.48 (0.22, 1.06)
Crohn’s disease	0.44 (0.23, 0.87)	0.41 (0.20, 0.84)

## Data Availability

Upon reasonable request, data will be provided by the corresponding author after consulting the IRB of KFMC.
